# Variations in entomological indices in relation to weather patterns and malaria incidence in East African highlands: implications for epidemic prevention and control

**DOI:** 10.1186/1475-2875-7-231

**Published:** 2008-11-04

**Authors:** Mojca Kristan, Tarekegn A Abeku, James Beard, Michael Okia, Beth Rapuoda, James Sang, Jonathan Cox

**Affiliations:** 1 London School of Hygiene & Tropical Medicine, Keppel Street, London, WC1E 7HT, UK; 2 National Malaria Control Programme, Ministry of Health, P O Box 7272, Kampala, Uganda; 3 Division of Malaria Control, Ministry of Health, P O Box 20750, Nairobi, Kenya

## Abstract

**Background:**

Malaria epidemics remain a significant public health issue in the East African highlands. The aim of this study was to monitor temporal variations in vector densities in relation to changes in meteorological factors and malaria incidence at four highland sites in Kenya and Uganda and to evaluate the implications of these relationships for epidemic prediction and control.

**Methods:**

Mosquitoes were collected weekly over a period of 47 months while meteorological variables and morbidity data were monitored concurrently. Mixed-effects Poisson regression was used to study the temporal associations of meteorological variables to vector densities and of the latter to incidence rates of *Plasmodium falciparum*.

**Results:**

*Anopheles gambiae *s.s. was the predominant vector followed by *Anopheles arabiensis*. *Anopheles funestus *was also found in low densities. Vector densities remained low even during periods of malaria outbreaks. Average temperature in previous month and rainfall in previous two months had a quadratic and linear relationship with *An. gambiae *s.s. density, respectively. A significant statistical interaction was also observed between average temperature and rainfall in the previous month. Increases in densities of this vector in previous two months showed a linear relationship with increased malaria incidence.

**Conclusion:**

Although epidemics in highlands often appear to follow abnormal weather patterns, interactions between meteorological, entomological and morbidity variables are complex and need to be modelled mathematically to better elucidate the system. This study showed that routine entomological surveillance is not feasible for epidemic monitoring or prediction in areas with low endemicity. However, information on unusual increases in temperature and rainfall should be used to initiate rapid vector surveys to assess transmission risk.

## Background

The highlands of East Africa are characterized by unstable malaria transmission and local populations typically have little or no immunity to the disease. Epidemics remain a significant public health issue in these areas, resulting in significant morbidity and mortality [1].

There is a well-recognized need to develop malaria early warning and detection systems in order to avert or reduce the negative impacts of epidemics in highland areas [2,3]. For such systems to be effective, the lead times they provide must be sufficient to allow for increased surveillance, planning of prevention and control measures and targeting of specific areas [4]. In the past, researchers have attempted to build early warning models based on observed or predicted climate anomalies [5-8]. This approach has shown some promise in certain epidemiological settings [7], but progress in developing climate-based models of malaria incidence in highland areas has been very limited. Although past highland malaria epidemics have been shown to occur within defined altitudinal limits [9] and have often been linked causally to climatic anomalies [10,11], scientific knowledge of interactions between climate, vectors and disease has not yet reached a stage where it can directly contribute to the design of early warning systems. Vector density has been proposed as a suitable indicator of epidemic risk [12], but its practical use within routine monitoring systems has not been evaluated.

To provide a basis for modeling transmission in these areas, temporal variations in vector densities and human biting rates were investigated in relation to changes in meteorological variables and malaria incidence at four highland sites in Kenya and Uganda. On this basis, it was also possible to assess the utility of *Anopheles *indoor resting density as an indicator for epidemic monitoring. Practical implications of the study in relation to vector control measures and epidemic prevention in highland areas are discussed.

## Materials and methods

### Study areas

**Figure 1:**
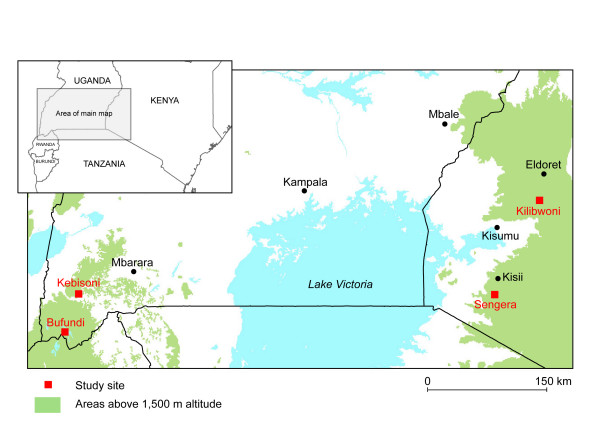
Map showing locations of the four study sites in Uganda and Kenya.

Locality-specific meteorological, entomological and malaria morbidity data were collected within the Highland Malaria Project [4] at four health centres located at varying altitudes (Table 1): Bufundi and Kebisoni in south west Uganda, and Sengera and Kilibwoni in western Kenya (Figure 1). All localities are rural and the livelihood of the populations is based largely on subsistence agriculture. In Bufundi, adult males also work as seasonal labourers in mechanized farms in other districts.

**Table 1:** Altitude, annual climate and malaria incidence at Bufundi and Kebisoni, Uganda, and Sengera and Kilibwoni, Kenya.

Site	Altitude (m)	Average temperature (°C)			Average annual rainfall (mm)	Malaria incidence rate per 1000 per year*
				
		Minimum	Maximum	Mean		
Bufundi, Uganda	2291	12.8	21.4	16.1	884	15.6
Kebisoni, Uganda	1670	15.5	26.4	20.1	1,007	359.8
Sengera, Kenya	1816	14.4	25.8	18.9	1,709	3.4
Kilibwoni, Kenya	2065	11.5	24.6	17.0	1,424	43.2

Reported climate data are for the period November 2002–November 2006 (Bufundi), November

2002–August 2006 (Kebisoni), February 2003–April 2006 (Sengera), and February 2003–September 2006 (Kilibwoni).

*All patients who have travelled during the 2 weeks prior to falling ill have been excluded from the analyses.

Bufundi Health Centre is located in Kishanje parish, Kabale District, Uganda (1°17'S, 29°52' E; elevation 2291 m). Kishanje has a steep topography and most people in this area live in valleys within approximately 50–150 m below the level of the health centre. Nevertheless, this remains the highest and, therefore, the coolest of the four study sites. Average temperatures are fairly constant throughout the year, with slight peaks occurring in February, August and September. Rain falls in all months except June and July, with two peaks: the main one being in April and a second in September.

Kebisoni Health Centre is located in Kakinga parish, Rukungiri District, Uganda (0°51' S, 30°0.9' E; elevation 1670 m). Kakinga parish is characterized by a mild climate with two rainy seasons: March-April and September-November. Temperatures are highest in February and between June and August.

Sengera is the only non-governmental health centre among the four sites and is located in Bosoti sub-location, Gucha District, Kenya (0°52'S, 34°43'E; elevation 1816 m). There are several local tea plantations consisting of small holdings owned by local farmers. The hottest months are March and November, and the two rainy seasons are April-June and September-December.

Kilibwoni Health Centre (in Kilibwoni sub-location) is located in North Nandi District, Kenya (0°13'N, 35°14'E; elevation 2065 m). The cool and wet climate is suitable for growing tea, which is a significant cash crop in the district. Peak temperatures occur during February and March. There are two rainy seasons: March-May and July-September.

Except Kishanje in Bufundi, altitudes of the villages around the other three health centres where entomological studies were carried out are located within approximately similar altitudes to the health centres themselves.

### Anopheline sampling

Mosquitoes were collected at each site between 06.00 and 09.00 hours, at weekly intervals, for 47 months, using the pyrethrum spray sheet collection method [13]. Twelve houses were selected at the beginning of the study in the same parishes (Uganda) or sub-locations (Kenya) as the health centres. Preference was given to houses in the vicinity of breeding sites during the sample selection. Mosquito sampling took place in the same houses throughout the study except in a few instances where changes were necessary due to reasons such as abandoning of the houses by occupants, in which case an adjacent house replaced the original one. Verbal consent was sought from the head of each household at the start of the study.

Vector species were first morphologically identified and the blood digestion stage for each mosquito was recorded. Specimens were stored dry on silica gel pending further analysis. Members of the *An. gambiae *complex and *An. funestus *group were identified to the species level using the polymerase chain reaction method (PCR) [14,15]. The precipitin ring test [16] was used for blood meal analysis to test freshly fed and half-gravid mosquitoes for human, bovine and goat blood. Infection rates were determined using the sporozoite ELISA [17].

### Meteorological data

Outdoor temperature, relative humidity and rainfall data were recorded on a half-hourly and daily basis using solar-powered automatic weather stations (Campbell Scientific Limited, Loughborough, UK), installed within the compounds of each health centre. Missing values for rainfall resulting from blocked rain gauges (and constituting 4.5% of total observations for this period) were imputed using linear interpolation [18].

### Parasitological data

At each site, of all the patients who attended the health centres for routine treatment, only those patients diagnosed with clinical malaria according to standard national procedures for case management were subsequently sent to the health facility's laboratory for confirmation using a rapid diagnostic test (RDT) for *P. falciparum *(Paracheck Pf^®^, Orchid Biomedical Systems, Goa, India), and all other patients were not subject to the diagnostic procedure. All patients diagnosed clinically as malaria cases were given full treatment irrespective of the outcome of the RDT. Ethical approval for the study was obtained from relevant authorities in each country and the London School of Hygiene and Tropical Medicine. Only malaria cases from the parish or sub-location where entomological data were collected were included in the analysis. All patients who have travelled during the two weeks prior to diagnosis were excluded from the analyses.

### Data analysis

Due to low vector densities, analysis was based on pooled monthly data for each site. For each vector species the indoor resting density (*IRD*) was calculated as the number of mosquitoes per house per day. The human biting rate (*HBR*) was calculated from the indoor collections using: *HBR = (F+H)/(2N)*, where *F = *number of freshly fed mosquitoes, *H = *number of half-gravid mosquitoes, and *N = *number of people who slept in the study house during the previous night. The human blood index (*HBI*), defined as the proportion of freshly-fed and half-gravid mosquitoes testing positive for human blood, and the sporozoite rate were determined for each vector species. Temporal variations in vector species composition and indoor density, human biting rates and sporozoite rates during the study period were analysed in relation to site-specific meteorological and malaria incidence data. The confounding effects of wall and roof types of the sampled houses were controlled for during the analysis involving differences in vector density between the sites. Microsoft Access Version 2000 (Microsoft Corporation, Seattle, USA) was used for data entry and Stata Version 10 (StataCorp, College Station, Texas, USA) was used for data analysis. Mixed-effects Poisson regression was used to study the temporal associations of meteorological variables to vector densities and of the latter to incidence rates of *P. falciparum *at the four sites. In both cases, forward regression was used to enter independent variables up to lags of two months (i.e. lags 1 and 2). The models take into account serial correlations in the longitudinal data and inter-area variations. Due to low numbers of *An. arabiensis *and *An. funestus*, the models were developed for the predominant vector *An. gambiae *s.s.

## Results

### Climatic factors and morbidity

**Figure 2:**
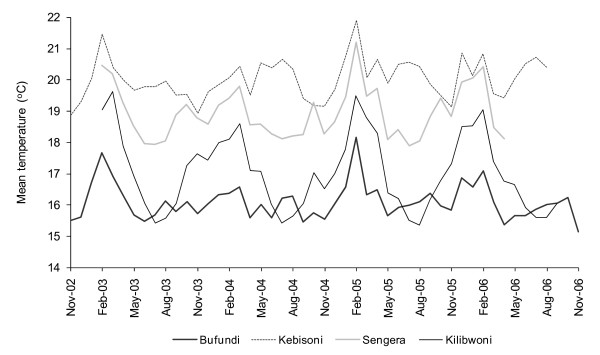
Variations in mean temperature in sentinel epidemic surveillance sites in Kenya and Uganda, November 2002 – November 2006.

Although clear differences exist in terms of average climate conditions (Table 1), temporal weather patterns were broadly consistent across the four sites (Figure 2). For example, unusually low levels of relative humidity and high temperatures were observed in all sites in February 2003 and February 2005. Rates of malaria incidence varied between sites and, with the exception of Sengera, appeared to be negatively correlated with altitude (Table 1). Sengera, a non-governmental health centre, is the only facility which routinely charges patients fees for treatment services and this seems to be reflected in the relatively low number of malaria cases presenting at this site.

### Anopheline species composition

*Anopheles gambiae *s.s., *An. arabiensis *and *An. funestus *were found at all sites, except in Bufundi where *An. arabiensis *was absent (Table 2). *Anopheles gambiae *s.s. was the predominant vector species in all sites except in Kilibwoni, where *An. arabiensis *was the most common. *Anopheles funestus *was generally the least abundant of the three species except in Kebisoni, where it was found more frequently than *An. arabiensis*. Other non-vector anopheline species recorded included *Anopheles leesoni*, *Anopheles parensis *and *Anopheles christyi*.

**Table 2:** *Anopheles *species composition at Bufundi and Kebisoni, Uganda, and Sengera and Kilibwoni, Kenya.

Site	Number of *Anopheles *mosquitoes collected			
	
	*An. gambiae *s.s.	*An. arabiensis*	*An. funestus*	Other
Bufundi (2291 m)	3	0	1	2
Kebisoni (1670 m)	169	5	9	16
Sengera (1816 m)	21	3	1	5
Kilibwoni (2065 m)	17	27	2	118

### Vector density

Densities of anopheline vectors were generally low in all sites, including during malaria seasons. The highest density of *An. gambiae *s.s. (0.79 mosquitoes/house/day) was recorded in Kebisoni in December 2004. Densities of *An. arabiensis *were generally lower than those of *An. gambiae *s.s., except in Kilibwoni where it reached a peak (0.12 mosquitoes/house/day) in April 2003. The density of *An. funestus *(0.05/house/day or less) was lower than those of the other two vectors. Its density usually peaked after the decline in density of the other two vector species. The highest average vector density of 0.08 mosquitoes/house/day was recorded at Kebisoni, a site with the lowest altitude and the highest malaria incidence. At Bufundi, the highest of the four sites, vector density was the lowest (0.002 mosquitoes/house/day).

A linear regression model was used to control for the possible bias that might have been introduced as the result of imbalance between the four sites in terms of the number of sampled houses with different wall and roof types. Four types of houses were included in the samples: Type A (mud walls and metal roofs), Type B (mud walls and thatched roofs), Type C (a mixture of cement and mud walls and metal roofs), and Type D (wooden walls and metal roofs). Ten of the 12 sampled houses in Bufundi, 10 in Sengera and 11 in Kebisoni were Type A, and the rest were Type B. The Kilibwoni sample included all house types: A (1), B (6), C (4), and D (1). Variation in vector density between the sites was significant even when the effect of wall and roof types was taken into account. Within each site, there were some preferences of mosquitoes for certain types of houses. However, the preferences varied between sites. For example, higher densities of *An. gambiae *s.s. were collected in Kebisoni (altitude 1670 m) in houses with mud walls and metal roofs compared to houses with mud walls and thatched roofs, whereas in Kilibwoni (altitude 2065 m) the opposite was true. These results indicate that within a site, proximity to breeding sources was more important as a determinant of density rather than the type of houses sampled.

### Human blood index and human biting rate

*Anopheles gambiae *s.s. was the most antropophagic species and *An. arabiensis *the least antropophagic species (Table 3). *HBR *at all four sites was below one bite/person/night in any given month during the study.

**Table 3:** Origin of blood meals and human blood index (HBI) of mosquitoes collected indoors at Bufundi and Kebisoni, Uganda, and Sengera and Kilibwoni, Kenya.

Site	Vector species	No. tested	Human	Bovine	Goat	Unknown	HBI*
Bufundi	*An. gambiae *s.s.	3	2	0	1	0	0.67

Kebisoni	*An. gambiae *s.s.	114	89	2	1	22	0.78
	*An. arabiensis*	3	1	0	0	2	0.33
	*An. funestus*	9	5	0	0	4	0.56

Kilibwoni	*An. gambiae *s.s.	12	7	0	3	2	0.58
	*An. arabiensis*	18	3	9	4	2	0.17
	*An. funestus*	1	0	0	0	1	0.00

Sengera	*An. gambiae *s.s.	16	9	0	1	6	0.56
	*An. arabiensis*	2	1	0	0	1	0.50
	*An. funestus*	1	1	0	0	0	1.00

Total	*An. gambiae *s.s.	145	107	2	6	30	0.74
	*An. arabiensis*	23	5	9	4	5	0.22
	*An. funestus*	11	6	0	0	5	0.55

*HBI = Human Blood Index

### Sporozoite rate and entomological inoculation rate (EIR)

Over the study period as a whole, the sporozoite rate for *An. gambiae *s.s. was 5.9% (1/17) at Kilibwoni and 1.2% (2/169) at Kebisoni. *EIR *was greater than zero only in February 2004 at Kilibwoni (0.09 infective bites/person/month) and in December 2004 at Kebisoni (0.04 infective bites/person/month).

### Seasonality of climate variables, morbidity and entomological variables

Rainfall was seasonally bimodal in all sites except Bufundi. Levels of relative humidity typically increased following the rainy seasons and average temperatures peaked twice each year in all sites except at Kilibwoni.

At Bufundi peaks in the number of malaria cases were recorded during June and again during December, 2–3 months after the peaks in rainfall.

Like Bufundi, Kilibwoni is located at an altitude above 2000 m and has a similarly low incidence of malaria, despite experiencing higher vector densities. A peak in rainfall in April-May was followed by a distinct peak in malaria incidence in June. However, the second peak in rainfall in August was preceded by a decrease in temperature in July, and was followed by a smaller peak in incidence in October. *Anopheles arabiensis *was the predominant species present throughout most of the year, whereas *An. gambiae *s.s was present only from January to July (albeit in relatively high densities), and *An. funestus *appeared in January and October outside the rainy seasons.

The highest malaria incidence was observed at Kebisoni, which is the warmest of the four sites, where vector abundance was also the highest. *Anopheles gambiae *s.s. was present almost throughout the year with two peaks in April and December, during the rainy seasons. *Anopheles arabiensis *was found only in May and November, whereas *Anopheles funestus *was collected only between March and May.

**Figure 3:**
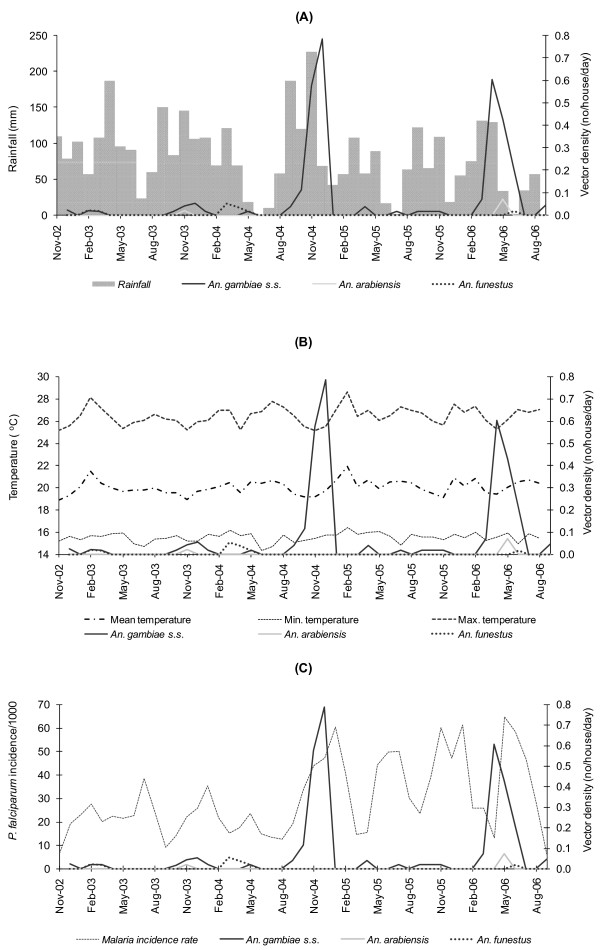
Temporal fluctuations in vector densities in relation to (A) rainfall, (B) mean, minimum and maximum temperatures and (C) *P. falciparum *incidence rates, at Kebisoni, Rukungiri District in Uganda (November 2002–September 2006).

Of the four sites, Sengera experienced the lowest rates of malaria incidence based on available passive case detection data. Peaks in the indoor resting densities of the three vectors were preceded first by an increase in temperature and then by a peak in rainfall. This pattern was also observed at Kebisoni. Except in a few cases, in general the peaks in vector densities appear to be followed by peaks in malaria incidence after 1–2 months (Figure 3C).

### Temporal associations of climatic, entomological and morbidity variables

The two-level mixed-effects Poisson regression analyses using all the data collected at the four sites showed that average temperature in previous month and rainfall in the previous two months had a quadratic and linear relationship with *An. gambiae *s.s. density, respectively, whereas increases in densities of this vector in previous two months showed a linear relationship with increased malaria incidence (Tables 4 and 5). The statistical interaction term between average temperature and rainfall in the previous month was highly significant. Adding average relative humidity in previous two months did not significantly improve the goodness-of-fit of the model for *An. gambiae *s.s. density.

**Table 4:** Estimates of the fixed parameters of mixed-effects Poisson regression to study the relationships between meteorological variables and ln(*An. gambiae *s.s. densities per house per day) (*N *= 160; number of groups = 4).

Effect	Estimate (S.E.)	*p*	95% confidence interval	
Average temperature in the previous month	11.560 (2.8288)	<0.0001	6.016	17.105
Average temperature in the previous month squared	-0.329 (0.0744)	<0.0001	-0.475	-0.183
Rainfall in the previous month	-0.055 (0.0273)	0.045	-0.108	-0.001
Rainfall at lag of two months	0.007 (0.0015)	<0.0001	0.004	0.010
Interaction term: average temperature in the previous month × rainfall in the previous month	0.003 (0.0014)	0.015	0.0007	0.0063
Constant	-107.609 (26.9111)	<0.0001	-160.353	-54.864

**Table 5:** Estimates of the fixed parameters of mixed-effects Poisson regression to study the relationships between *An. gambiae *s.s. densities and ln(*P. falciparum *incidence rates per 1000 population per month) (*N *= 164; number of groups = 4).

Effect	Estimate (S.E.)	*p*	95% confidence interval	
*An. gambiae *s.s. density/house/day during the previous month	0.983 (0.061)	<0.0001	0.863	1.103
*An. gambiae *s.s. density/house/day at lag of two months	0.471 (0.067)	<0.0001	0.341	0.602
Constant	-6.045 (0.861)	<0.0001	-7.732	-4.357

At Kebisoni, there was a dramatic increase in *An. gambiae *s.s. vector density in November and December 2004 (on average 0.68 mosquitoes/house/day, or approximately 2 females in 3 houses per day). This increase was preceded by unusually high rainfall between September and November 2004 (Figure 3A) and by an increase in temperature in July and August 2004 (Figure 3B). The unusual increase in *An. gambiae *s.s. density in November and December 2004 was followed by an approximately two- to three-fold increase in malaria incidence in January 2005, compared to what would have been expected for that month. This was classified as an outbreak period, as determined by the method developed by Abeku and colleagues [4], based on about 10 years clinical data compiled for the site.

A second marked increase in *An. gambiae *s.s. density at Kebisoni was observed in April and May 2006 (on average 0.52 mosquitoes/house/day or one female in two houses per day) following increases in rainfall in February and March 2006 (Figure 3A) and in temperature in November and December 2005 (Figure 3B). A three- to four-fold increase in malaria incidence compared to the expected level was observed in May 2006 following the increase in vector density (Figure 3C). In contrast, increases in malaria incidence at the same site during July 2005 and November 2005–January 2006 were not preceded by unusual increases in rainfall and vector density, although temperature was above what would be normally expected during these months.

At Kilibwoni, the period between February and April 2003 can be classified as being a malaria outbreak period based on baseline clinical data of approximately eight years. *Anopheles gambiae *s.s. and *An. funestus *were found in January 2003, and the density of *An. arabiensis *started to rise suddenly in February, reaching a peak in April 2003. This peak in density followed an increase in temperature one month earlier. However, no abnormal rainfall pattern was observed.

An epidemic period at Sengera in January and February 2003 coincided with a peak in *An. gambiae *s.s density (0.27 mosquitoes/house/day). Rainy seasons in 2004 and above-average temperatures during those periods coincided with resurgence of vectors, particularly *An. gambiae *s.s., and an increase in number of malaria cases.

## Discussion

This study indicates that increases in malaria incidence (occasionally to epidemic levels) in the highlands follow relative increases in the normally low densities of the local vectors, which in turn, are preceded by changes in meteorological variables including temperature and rainfall. Malaria transmission is unstable at high altitudes and can result in epidemics whenever weather conditions favor transmission [19]. The ability of vectors to survive and breed at high altitudes has been questioned and the presence of malaria in the African highlands has often been attributed to the introduction of vectors from nearby lowlands [20,21]. Garnham [20] described malaria epidemics occurring at altitudes of 2200–2500 m at Londiani, Kenya, where the presence of *An. gambiae *was confirmed. It was thought they were introduced into the area annually and only managed to breed for a short period in May. More recently, *An. gambiae *s.l. was found in very low densities in Kabale District, Uganda, up to an altitude of 2400 m [12].

In this study, health facility-based passive case detection data were used to estimate fluctuations in incidence in the community. Although this approach probably underestimates the true incidence of malaria, it is reasonable to assume that relative changes in malaria case numbers recorded at health facilities mirror those of malaria incidence in the local population, provided no significant changes in utilization at individual sites occur over the same period. In reality, there will always be "artefacts" within surveillance records that are not related to changes in malaria transmission, and these have the potential to confound analyses of this type. Changes in government policy regarding payments and subsidies, for example, will have significant effects on service utilization. In the context of this analysis, malaria treatment fees were waived early on in the study in all government sites in both countries. However, patients continued paying fees at non-governmental facilities, which means that outpatient attendances in the latter were comparatively low. As an example, it is also not clear whether the low number of cases observed at Sengera, Gucha District, is a true indication of relatively low levels of malaria incidence in the local area, or a reflection of the fact that, as a non-governmental health centre, this is the only facility in the current study to charge user fees. This shows that surveillance systems for epidemic monitoring should largely be based on changes in malaria incidence over time rather than the incidence levels themselves. In this respect, caution is needed in interpretation of the results that compare incidence levels among the four sites and how they were affected by various entomological indices.

Vector densities were generally low in all the four sites, including during malaria seasons. Similar findings have been described in other studies in the East African highlands [11,22-24]. Differences in vector density observed between the four sites were due to their variation in altitude, even when the possible bias due to types of houses sampled was taken into account. *Anopheles *density of 0.25 females/house/day has been reported as a critical density indicating epidemic risk [12]. In the present study, vector densities higher than 0.25 females/house/day were observed before some of the epidemic episodes, although there were exceptions. There are, however, a number of difficulties associated with using *Anopheles *density as an indicator of epidemic risk. Densities are often very low in the highlands, which means frequent sampling or large sample sizes are required for this to be a sensitive indicator. Lack of prior information on mosquito distribution and "normal" density and difficulties in determining what constitutes an adequate sample for assessing "significant" changes and detecting the critical density also pose problems [12,24]. In highland areas, the basic reproduction number of malaria (*R*_*0*_) is typically below 1 during non-epidemic periods. At the threshold value of *R*_*0 *_= 1, the "critical" vectorial capacity (C*) is equal to the recovery rate (*r*). Vectorial capacity is a function of vector density in relation to humans (*m*), the daily frequency of feeding on humans (*a*), the daily survival probability of the vector (*p*), and the length of the sporogonic period (*n*) [25]. If C* = r, then it can be shown that the "critical" vector density (m*) is m* = -*r*ln*p*/(*a*^2^*p*^*n*^) [26]. It is well known that *p*, *a *and *n *depend to a large extent on environmental temperature [27-30]. The daily survival probability also depends on humidity and varies between vector species [31]. The recovery rate *r *can vary between areas depending on immunity levels and effectiveness of treatment interventions. The critical vector density will, as a result, vary significantly from area to area and cannot be used as a universal indicator for epidemic early warning.

The statistical model used in the present study revealed that temperature and rainfall in the previous month and rainfall at lag of two months were significantly associated with *An. gambiae *s.s. density of the current month. As expected, temperature had a quadratic effect and its interaction with rainfall was highly significant. Larval development rates are dependent on temperature, which would determine whether or not excess rainfall leads to the formation of productive larval habitats. Higher temperatures would result in accelerated development of the aquatic stages before rain pools dry up. These results confirm the importance of monitoring both temperature and rainfall in highland areas for malaria early warning. Ideally, unusual changes in weather conditions should prompt health services to carry out rapid entomological surveys to assess the risk of increased transmission. For example, rapid surveys of larval or adult vector densities can be useful in epidemic early warning once abnormal weather conditions are observed. However, to decide which levels of meteorological variables are considered abnormal requires detailed area-specific investigation of historical weather patterns and their association with past epidemic events. Nevertheless, the present study also shows that the routine use of entomological studies will not be cost-effective because of large sample sizes required to detect presence of vectors and the associated cost.

High levels of *P. falciparum *prevalence can exist at very low levels of transmission by local vectors, even with very low densities and levels of *EIR*. Because vectors are relatively scarce in highlands and sporozoite rates are low, it is not only difficult to determine *EIR*, but *EIR *itself is of little use in measuring temporal variations in transmission in these areas. The *HBI *values for the three vector species in the present study were also lower than those recorded in lowlands of western Kenya [32] and at the Kenyan coast [33].

The results of this study suggest that relationships between different meteorological factors, altitude and vector density are complex and, to a degree, site specific. Conflicting findings have been reported on the association between rainfall, entomological variables and transmission [11,34,35]. Moreover, despite recent advances [6], malaria prediction and early warning systems cannot yet be used to provide sufficiently accurate malaria forecasts in highland areas. Therefore, more detailed analysis will be required and suitable mathematical models need to be developed to fully understand the importance of different factors in the genesis of epidemics in these and similar highland areas. This study provides the basis for developing such a model, which in turn, will be used to develop improved appropriate epidemic prediction systems for these areas.

The findings of this study also imply the need to implement different vector control strategies in different highland areas. It has been proposed that indoor residual spraying (IRS) interventions should mainly target areas that are sources of transmission bordering the highlands [36]. In Uganda, de Zulueta and colleagues [37] carried out mass drug administration and spraying operations within the area around Lake Bunyonyi in Kabale District where *An. funestus *breeds, only up to an altitudinal limit of 7000 ft (2134 m). Houses left unsprayed above that limit were effectively protected. During the period of the present study, the coverage of insecticide-treated nets (ITN) in the study areas was low. IRS was occasionally carried out, especially as a late response to an epidemic [38]. At very high altitudes, such as parts of Bufundi, with extremely low vector densities, IRS is unlikely to be the most appropriate option [39]. However, barrier spraying of neighboring valleys could prove useful to prevent widespread transmission of malaria, as these are usually the sources of vector breeding sites [36,39]. In epidemic situations, mass (fever) treatment using relatively inexpensive artemisinin-based combination therapy (ACT) drugs is recommended. This requires careful monitoring of the increase in malaria cases. In lower lying areas such as Kebisoni, targeted IRS once a year may be an appropriate measure around areas that represent transmission sources. The present study showed that routine entomological surveillance is not feasible for prediction of epidemics in areas with low endemicity such as the highlands of Kenya and Uganda. However, information on unusual increases in temperature and rainfall should be used to initiate a rapid vector survey in order to assess the risk of transmission in epidemic-prone areas. Targeted preventive interventions are effective only where an extended transmission is anticipated and rapid deployment of resources is possible. Close weekly monitoring of disease trends and strengthening preparedness, especially by stocking adequate antimalarials, should follow observed or predicted abnormal weather changes or increased vector densities in highland areas to initiate rapid interventions against potential epidemics.

## Competing interests

The authors declare that they have no competing interests.

## Authors' contributions

TAA, MK and JC designed the study and supervised the staff; MK and TAA trained the field staff; MO supervised the field study in Uganda; BR and JS supervised the field study in Kenya; JB designed and maintained the database used in the study, supervised field staff and commented on the manuscript; MK processed entomological specimens in the laboratory; TAA and MK compiled the data and carried out statistical analyses. MK wrote the manuscript; TAA and JC co-wrote the manuscript. All authors (except BR who sadly passed away) read and approved the final version.
